# Dr. Francis L. Haynes

**Published:** 1898-12

**Authors:** 


Dr. Francis L. Haynes, of Los Angeles, Cal., died at his home on Tuesday, October 18, 1898, aged 48 years. He was an alumnus of the University of Pennsylvania, class of '70, and was engaged in the practice of his profession at Philadelphia for several years. He removed to Los Angeles in 1887 on account of a threatened pulmonary disease. Soon after he was elected professor of gynecology in the Medical college of the University of Southern California, and, notwithstanding his continued ill health, he soon became recognised as one of the most skilful and scientific surgeons and physicians on the Pacific coast. In surgery, he was the apostle of antisepsis in Southern California, and by his untiring efforts did much to introduce and encourage here surgical Listerism, or surgical cleanliness. He was credited with being the first in Southern California to perform several of the most important radical operations known to modern surgery. His friends were many and their grief was keen when the end came to this distinguished physician.

